# Increasing Engagement of Women Veterans in Health Research

**DOI:** 10.1007/s11606-021-07126-4

**Published:** 2022-03-29

**Authors:** Joya G. Chrystal, Karen E. Dyer, Cynthia E. Gammage, Ruth S. Klap, Diane V. Carney, Susan M. Frayne, Elizabeth M. Yano, Alison B. Hamilton

**Affiliations:** 1 VA HSR&D Center for the Study of Healthcare Innovation, Implementation and Policy (CSHIIP), VA Greater Los Angeles Healthcare System, Los Angeles, CA USA; 2 Department of Psychiatry and Biobehavioral Sciences, UCLA David Geffen School of Medicine, Los Angeles, CA USA; 3 VA HSR&D Center for Innovation to Implementation (Ci2i), VA Palo Alto Health Care System, Menlo Park, CA USA; 4 Division of Primary Care and Population Health, Stanford University School of Medicine, Stanford, CA USA; 5 Department of Health Policy and Management, UCLA Fielding School of Public Health, Los Angeles, CA USA

**Keywords:** patient engagement, research engagement, Veterans, vulnerable populations, women’s health

## Abstract

**Background:**

Meaningful engagement of patients in health research has the potential to increase research impact and foster patient trust in healthcare. For the past decade, the Veterans Health Administration (VA) has invested in increasing Veteran engagement in research.

**Objective:**

We sought the perspectives of women Veterans, VA women’s health primary care providers (WH-PCPs), and administrators on barriers to and facilitators of health research engagement among women Veterans, the fastest growing subgroup of VA users.

**Design:**

Semi-structured qualitative telephone interviews were conducted from October 2016 to April 2018.

**Participants:**

Women Veterans (*N*=31), WH-PCPs (*N*=22), and administrators (*N*=6) were enrolled across five VA Women’s Health Practice-Based Research Network sites.

**Approach:**

Interviews were audio-recorded and transcribed. Consensus-based coding was conducted by two expert analysts.

**Key Results:**

All participants endorsed the importance of increasing patient engagement in women’s health research. Women Veterans expressed altruistic motives as a personal determinant for research engagement, and interest in driving women’s health research forward as a stakeholder or research partner. Challenges to engagement included lack of awareness about opportunities, distrust of research, competing priorities, and confidentiality concerns. Suggestions to increase engagement include utilizing VA’s patient-facing portals of the electronic health record for outreach, facilitating “warm hand-offs” between researchers and clinic staff, developing an accessible research registry, and communicating the potential research impact for Veterans.

**Conclusions:**

Participants expressed support for increasing women Veterans’ engagement in women’s health research and identified feasible ways to foster and implement engagement of women Veterans. Given the unique healthcare needs of women Veterans, engaging them in research could translate to improved care, especially for future generations. Knowledge about how to improve women Veterans’ research engagement can inform future VA policy and practice for more meaningful interventions and infrastructure.

## BACKGROUND

The engagement of patients in research as equitable partners is an increasingly recognized tenet of health services research. Patient engagement in research is defined as “the active, meaningful, and collaborative interaction between patients and researchers across all stages of the research process, where research decision making is guided by patients’ contributions as partners, recognizing their experiences, values, and expertise.”^1^ Various patient engagement frameworks describe the context and function of patients as stakeholders; involvement occurs along a continuum of pathways whereby patients advise, inform, or partner in research.^2–4^ Despite the current momentum to equip health research with patient stakeholders, the concept of research engagement is less familiar to patient populations, and therefore met with its own unique challenges for adoption and inclusion within health research practice.^5–7^

Recent works have demonstrated the added value of engagement for healthcare organizations,^8^ stakeholders,^9,10^ and specific groups of patients such as Veterans,^11,12^ including successful implementation of engagement resources^13^ and the influence of engagement on policy.^14^ The Veterans Health Administration (VA) has committed to patient-centered care,^15^ building on findings that individuals who actively participate in their healthcare are more satisfied, and have better outcomes at reduced costs—i.e., the “triple aim” of healthcare systems.^16^ Promoting patient engagement in health research may be especially useful in improving quality of care among high-risk or under-represented populations, such as women Veterans.^17^

Women’s increased enrollment in the military is reshaping the Veteran population. The fastest growing group of eligible VA healthcare users are now women.^18,19^ This trend has been accelerated by increased enrollment of women Veterans from recent wars in Iraq and Afghanistan.^20^ Women’s military experiences, and their responses to those experiences, are often distinct from men’s; these distinctions can affect healthcare needs and outcomes. Given women Veterans’ unique experiences and care needs, ^21^ participation in health research could result in findings that have the potential to directly (positively) impact their own healthcare.

This qualitative study is the first to explore the concept of engagement in research from the perspectives of women Veteran patients, VA women’s health primary care providers (WH-PCPs), and administrators. Women Veterans’ high rate of attrition from VA care,^22,23^ combined with persistent organizational barriers to care,^24^ highlight the need for VA infrastructure to leverage impactful research. Achieving more equitable healthcare, grounded in research, requires a deeper understanding of women’s engagement challenges and recommendations from multilevel perspectives. This paper aims to (1) examine the perspectives of women Veterans, WH-PCPs, and administrators on women Veterans’ research engagement, specifically barriers to and facilitators of engagement, and (2) identify ways to foster increased research engagement among women Veterans.

## METHODS

### Study Design, Setting, and Sample

Qualitative, semi-structured telephone-based interviews were conducted with women Veterans, WH-PCPs, and administrators, across five geographically dispersed VA medical centers (VAMCs) between October 2016 and April 2018. All sites are members of the VA Women’s Health Practice-Based Research Network (WH-PBRN), a national network of partnered VA facilities that supports the representation of women Veterans in VA research and quality improvement projects.^25^ Members of the study team worked with WH-PBRN Site Leads to coordinate local study recruitment efforts. To recruit key VA stakeholders in Women’s Health, WH-PBRN Site Leads provided study members with names and contact information for local WH-PCPs and site administrators (i.e., Women’s Health Medical Directors, Women Veteran Program Managers). WH-PCPs and administrators were sent email invitations by a study team member, and those interested were scheduled for interviews accordingly. To recruit women Veterans, Site Leads distributed study flyers at local VAMC women’s health clinics. Flyers described the study, participation incentive, and study contact information. Women Veterans phoned the study team to express interest, and upon confirming their Veteran status, were consented, and enrolled over the phone.

### Measures and Procedures

A semi-structured interview guide was developed and reviewed for comprehension. Interview questions were tailored for each sample group and sought perspectives on research experiences, and challenges to and facilitators of research engagement among women Veterans. A summary of interview guide questions is depicted in Table 1. All procedures were approved by the VA Central Institutional Review Board. Participants provided verbal consent to participation and audio-recording. Women Veterans were offered a $25 gift card for participation, whereas no study incentives were offered to WH-PCPs and administrators. Telephone interviews lasted on average 30 minutes. Demographic information was collected from women Veterans following interviews. Interviews were audio-recorded and professionally transcribed verbatim.

**Table 1:** Summary of interview guide

**Question categories**	
Experiences with/knowledge of research	
Attitudes/beliefs regarding engagement in research	
Preferred study role and level of engagement	
Practical issues (e.g., barriers, facilitators to participation)	
Interest in research engagement	
Engagement in women Veteran–related activities	
Views of women’s health research priorities in VA	

### Analysis

Deidentified transcripts were imported into ATLAS.ti (v7) for coding and analysis. Consensus-based coding was performed by two experienced qualitative analysts (JC, KD). In the first phase of coding, transcripts were reviewed, and thematic concepts were noted; this informed iterative development of a code list, which included *a priori* codes related to key topics of inquiry. Transcripts were coded using the constant comparative method,^26^ with discrepancies resolved through discussion. The study PI (AH) oversaw the coding process and reviewed coded transcripts for quality and consistency. Coded segments were analyzed to identify emergent themes.

## RESULTS

### Participants

Table 2 depicts the VAMC region and gender characteristics of VA stakeholders in Women’s Health (WH-PCPs and administrators). Women Veteran demographics are presented in Table 3. On average, women Veterans were 56 years of age, identified as single, and served during the Post-Vietnam era.

**Table 2:** VA Women's Health stakeholders (WH-PCPs and administrators)

	WH-PCPs (*N*=22)	Administrators (*N*=6)
**VAMC region**		
Great Lakes	13.6% (3)	16.7% (1)
South	9.1% (2)	33.3% (2)
Pacific Northwest	27.3% (6)	16.7% (1)
West	27.3% (6)	0.0% (0)
Midwest	22.7% (5)	33.3% (2)
**Gender**		
Male	4.5% (1)	33.3% (2)
Female	95.5% (21)	66.7% (4)

**Table 3:** Women Veteran demographics (*N*=31)

**VAMC region**	
Great Lakes	22.6% (7)
South	12.9% (4)
Pacific Northwest	22.6% (7)
West	19.3% (6)
Midwest	22.6% (7)
**Age, years**	
Mean (SD)	55.6 (13.0)
Range	25-86
**Race/ethnicity**	
White	45.2% (14)
African American	32.2% (10)
Other	9.7% (3)
Unknown	12.9% (4)
**Relationship status**	
Married, partnered	25.8% (8)
Single (including divorced, separated)	64.5% (20)
Unknown	9.7% (3)
**Children**	
Yes	41.9% (13)
No	48.4% (15)
Unknown	9.7% (3)
**Number of children**	
1	3.2% (1)
2	19.4% (6)
3	12.9% (4)
4	3.2% (1)
5	3.2% (1)
**Military branch**	
Army	29.0% (15)
Air Force	48.4% (9)
Navy	16.1% (5)
Coast Guard	3.2% (1)
Reserve/National Guard	3.2% (1)
**Era of service**	
During Vietnam Era	9.7% (3)
During Post-Vietnam Era	41.9% (13)
Between Persian Gulf War and 9/11	3.2% (1)
During September 11, 2001, to present	16.1% (5)
Extended service across multiple eras	19.4% (6)
Unknown	9.7% (3)
**Years served in military**	
Mean (SD)	8.14 (7.22)
Range	1.25–32.00

### Perceived Importance of Women’s Health Research

Women Veterans, WH-PCPs, and administrators overwhelmingly endorsed the importance of research for women Veterans’ health and healthcare. WH-PCPs emphasized the importance of driving sex- and gender-specific research forward to increase understanding and awareness of women Veterans’ unique healthcare needs. One WH-PCP stated: “I think it’s important to develop gender-specific care… If the armed services are accepting more women, they need to be able to say, ‘We welcome you and we have all of these wonderful resources for you through the VA.’” Some spoke about health research data (and related health treatments) as disproportionately focused on male civilian subjects: “We’ve been treating women as men, and we’re not.”

### Women Veterans’ Engagement Preferences and Experiences

None of the women Veterans reported having engaged in research as a patient stakeholder/partner previously. The vast majority were unfamiliar with the concept of patient research engagement, however expressed enthusiasm and interest for the inclusivity of patients to inform research beyond traditional subject roles. Moreover, most women Veterans reported little or no prior research experience. Half of women Veterans had never been invited to participate in health research (prior to the current study).

Women Veterans described factors that would be particularly helpful in their decision to engage in research: rationale for why the patient perspective is needed, clearly defined expectations and deliverables, relevant subject matter, and travel/time compensation details. Some expressed wanting to know personally relevant information about researchers in order to feel comfortable. For instance, a woman Veteran expressed a desire to know researchers’ personal motivations: “[I’d like to know] a little about the experience of the researcher... Are they from families where they had Veterans?… What motivated you?... What has been your experience with female Veterans?”

Women Veterans expressed altruistic motivations in their desire to engage in health research. A woman Veteran described her desire to help as a form of advocacy: “It was the idea that you’re helping the VA provide better care for the Veterans, particularly for female Veterans because there’s not very many females in studies.” Some said incentives were appreciated, but not a determining factor in their decision to participate. Other motivations included a desire to learn about a personally relevant topic, and interest in study outcomes following their participation; however, this feedback loop had not been closed for the minority who participated as research subjects.

### Engagement Barriers and Facilitators

Table 4 depicts women Veterans’, WH-PCPs’, and administrators’ converging and diverging perspectives on barriers to and facilitators of women Veterans’ engagement.

**Table 4:** Barriers to and facilitators of women Veterans’ research engagement

	Identified by women Veterans and WH-PCPs/administrators	Identified by women Veterans only	Identified by WH-PCPs/administrators only
**Barriers to women Veterans’ engagement**	• Unawareness of opportunities • Distrust of research • Competing priorities • Confidentiality concerns	• Reluctance to discuss military experiences • Belief that participation will not yield change	• Environmental concerns • Mental health distress
**Facilitators of women Veterans’ engagement**	• Utilization of patient-facing portals of the electronic health record • Warm hand-offs from provider/staff • Accessible research registry • Communicate potential research impact	• Outreach (e.g., social media, Veteran events)	• Research ambassadors • Provide Veterans with research findings • Trauma-informed research

### Convergent Barriers

Four convergent themes emerged across all participant groups as barriers to engagement: (1) unawareness of opportunities, (2) distrust of research activities, (3) competing priorities, and (4) confidentiality concerns.

#### Unawareness of Opportunities

Most of participants expressed that women are not aware of research opportunities. For instance, many women Veterans expressed the happenstance by which they learned about the current study (e.g., fellow woman Veteran passed along information, clerk provided a flyer). Some WH-PCPs were not clear whether research opportunities even existed within their VAMC, and surmised that women Veterans might be similarly unaware of such opportunities. An administrator shared that she would not know where to look for local research opportunities applicable to women Veterans: “Even working here, you know, it’s such a hard system to navigate. It’s such a maze.”

#### Distrust of Research

Distrust was identified as a pervasive barrier to research engagement. A woman Veteran explained: “I think we have a mistrust when it comes to, ‘Oh, somebody’s finally trying to do so something to help us when we’ve been struggling for such a long time’… We’ve been let down so much.” Some providers and administrators suggested that distrust of research may be correlated with former military experiences. A WH-PCP said: “There is distrust among female Veterans because of how they’ve been treated in the military system. Also, I guess I would feel the same way.” Another provider perceived research engagement as potentially too vulnerable an experience for women Veterans with a history of trauma: “Many of them have had previous assault… They’re going to be less likely to come into a place that is mirroring their military time.” The climate of distrust was noted as particularly salient for African Americans Veterans. An administrator said: “I think our African American population is truly suspicious of us doing research… Because of the Tuskegee issues, the atrocities of that, those suspicions have carried on generationally.” Another administrator made note of the various cultural complexities and biases that impact researchers: “There is a cultural bias against research among the African American community that is separate from the bias of a Veteran who has been in the system, that’s separate from a female Veteran and whatever experiences she may have had in the system that makes her biased against research.”

#### Competing Priorities

Participants suggested that women’s limited time and responsibilities to employers, children, and other dependents may take precedence. WH-PCPs recognized that adherence to medical care may already be a challenge for women Veterans compared to male Veterans; thus, they may be less likely to engage in voluntary commitments such as research. One WH-PCP said: “In general, women tend to put everybody first… A lot of taking care of their family, [which] leaves their health needs till last, but particularly for women Veterans.” Expanding scheduling options for research participation (e.g., by adding evening/weekend hours) may not be enough; as one women Veteran noted, “There is probably not enough weekend availability for women because they also function as parents or single mothers.”

#### Confidentiality Concerns

Women Veterans suggested there may be apprehension about data collection exposing something related to their military experience(s) that they would otherwise not want revealed (e.g., mental illness, military-related trauma). A woman Veteran shared: “They may be a little paranoid about confidentiality because they might have done things in their past that they don’t want to be revealed.” A few WH-PCPs and administrators theorized that women Veterans’ apprehension might stem from the belief that research might uncover health information that would jeopardize their VA benefits. A WH-PCP explained: “I think they would fear [that it] would affect their disability somehow, that it might affect their disability benefits, so it might be a sense of what good is it going to bring?”

### Divergent Barriers

Women Veterans suggested additional barriers not otherwise mentioned by WH-PCPs and administrators: (1) reluctance to discuss military experiences, and (2) belief that research participation will not yield change within VA. WH-PCPs and administrators identified two additional barriers to women Veterans’ engagement in research: (1) environmental concerns, and (2) mental health distress.

#### Reluctance to Discuss Military Experiences

Women Veterans suggested that other women Veterans may feel disinterested in research that may prompt them to explore the past, therefore making research engagement at VA less desirable. A woman Veteran explained this potential barrier, hinting at the discomfort one might feel if pressed to relay details or memories from the military: “I think that it’s hard for some women Veterans to be able to speak up due to some of the instances that does happen to them when they are in the service.”

#### Belief that Participation Will Not Yield Change

Women Veterans expressed that they were not convinced that participating in research would result in actual change. For instance a woman Veteran said, “I think they [women Veterans] truly don’t think anything will come of [research],” while another said, “Maybe they don’t think it’s going to change things.”

#### Environmental Concerns

WH-PCPs and administrators suggested that some women Veterans may decline participation due to their discomfort with less controlled environments on VA campuses outside of women’s clinics. For some women Veterans, particularly “those with [a history of] military sexual trauma,” the prospect of volunteering time outside the Women’s Health Clinic is not likely: “They feel more secure or less stressed in an area where it’s women…just less anxious in an area where it’s not full of men, male Veterans.” Another provider said: “Many of our patients don’t like being here, but the women especially don’t like being here… They try to avoid it.”

#### Mental Health Distress

According to WH-PCPs and administrators, mental health distress, particularly for those with anxiety and/or history of trauma, can impede women Veterans’ ability to engage in research activities. For instance, a WH-PCP said that the post-traumatic stress disorder (PTSD) symptoms among some women “would get in the way of actually volunteering.” Another provider discussed the unfortunate paradox of reaching this special population of women Veterans who “feel really alienated from the general larger system.”

### Convergent Facilitators

Four convergent themes emerged across all participant groups as facilitators of engagement: (1) electronic patient portal, (2) warm hand-off from provider-staff, (3) accessible research registry, and (4) communicate potential research impact.

#### Electronic Patient Portal

Participants recommended VA’s patient-facing portals of the electronic health record as a potential resource for advertising research engagement opportunities since many Veterans already use it as part of their routine healthcare. For instance, a woman Veteran suggested: “Put a [research] link for women Veterans… Technology is there, we just have to be creative and put it into place.” WH-PCPs had a similar endorsement: “Seem[s] to be the fastest and easiest way to get their attention.”

#### Warm Hand-Off from Provider/Staff

Participants suggested including providers or clinic staff as a potential strategy to improve engagement. WH-PCPs acknowledged that clinic staff may already feel overburdened; however, endorsements to connect to research from a trusted source is key for women Veterans: “It’s a familiar face presenting the research topic to the patient in a more comfortable setting, as opposed to a letter or phone call. [However] it would be difficult because I have yet to meet a primary care provider who is not overwhelmed.” Furthermore, an administrator pointed out that WHC staff are more familiar with their women Veteran patients (including their mental health needs), and therefore “good candidates to help researchers [engage patients].”

#### Accessible Research Registry

Some WH-PCPs and administrators referenced the usefulness of a registry to minimize research barriers: “A broker who can scan that database and give researchers the names of potential subjects, can then (under IRB approval) be contacted.” Another provider described the success of a local university that utilizes a registry: “It’s an opportunity for patients, [and] potential future research volunteers that either has health conditions or are normal healthy controls to opt in.” Although women Veterans did not mention the term “registry” or “repository” in their interviews, many suggested that the interviewer “keep my information on hand for future research opportunities.”

#### Communicate Potential Research Impact

Participants emphasized the importance of being transparent about a study’s purpose, privacy/confidentiality measures, and the potential impact or outcomes of the research. A WH-PCP underscored the importance of asserting that the goals of research would be to help other women Veterans: “…‘If you wish to participate, you would be helping other women Veterans.’ Usually, they are very amenable to taking part in it.” Women Veterans emphasized the importance of researchers’ communicating why their participation is needed, and how their involvement may result in helping other women Veterans.

### Divergent Facilitators

Women Veterans emphasized the importance of outreach to facilitate inclusion and engagement of women Veterans. WH-PCPs and administrators identified three additional facilitators to women Veterans’ engagement in research: (1) research ambassadors, (2) providing Veterans with research findings, and (3) trauma-informed research.

#### Outreach

Women Veterans suggested various avenues for outreach, including employing women Veterans to help disseminate information to other women Veterans. One woman explained: “We relate to one another. We trust one another, and we know that we are looking out for one another.” Without endorsements from women Veterans, a study team will need to “try to build authentic and genuine relationships” with the cohort they are interested in. For instance, some suggested VA research–sponsored events in order to build community relationships, and raise awareness about VA’s research mission. Some women suggested that research staff participate in local community events (e.g., VA Stand Downs), and to utilize social media forums to disseminate research opportunities.

#### Research Ambassadors

WH-PCPs and administrators suggested use of “research ambassadors” stationed in clinics or waiting rooms to help serve as a dedicated liaison to research. These ambassadors could function as informants (answer questions about research) as well as build rapport with the clinical community since research offices are often removed from clinical environments. For example, a WH-PCP suggested that the presence of research ambassadors would help to humanize research efforts and perhaps better engage women Veterans: “To have someone right there to talk with the patient can help with enrollment… They’re able to meet with the patient.”

#### Providing Veterans with Research Findings

WH-PCPs and administrators underscored the importance of connecting with participants following their involvement to provide study findings. An administrator explained that without researchers returning to inform others about their findings, the consequence may mean less research engagement in the future: “You never know what happened, where it went, what the aggregate results were. It goes off into space… ‘No thanks.’ It’s an hour of my time and it didn’t result in any change. You didn’t even tell me what the results were, and I think the patients feel that way too.”

#### Trauma-Informed Research

WH-PCPs and administrators emphasized the importance of integrating a trauma-informed lens when it comes to research engagement: “We need to do trauma-informed research to get at the heart of what we really need to do for them.” In the same way clinicians engage in trauma-informed patient care, researchers must learn how to integrate trauma-informed practices into their research design and delivery. An administrator said, “Trauma-informed research, it’s a very whole health type of perspective where we recognize exactly where they’re coming from and we’re able to design research projects that really speak to what’s important to them or to recognize them culturally, or what it is that they experienced. I think they experienced some pretty horrible things.”

## DISCUSSION

Although the engagement of Veterans in both patient care and health research capacities is becoming a standard of practice within VA and across other healthcare institutions,^9,27,28^ the concept of engaging in research as a stakeholder/partner was unfamiliar to the women Veterans in this study—most of whom had limited or no experiences participating in research as subjects, let alone as stakeholder/partner.

Participants expressed several reasons for why women Veterans may not engage in research, namely not knowing about opportunities and distrust of research. Previous studies acknowledge such challenges: both a lack of opportunity,^29^ and lack of trust between communities and research entities.^30,31^ Uncertainty about where to find research opportunities and its lack of visibility within the healthcare system may consequently create distance between researchers and women Veterans. Furthermore, the concept of trust (or lack thereof) is both ubiquitous and interrelated with other identified barriers including confidentiality and fear of exposure, which are particularly salient for Veteran populations.^13,32^ Trust is particularly complex because without it, a reciprocal relationship between women Veterans and researchers cannot exist. Krahe’s study on perceived risk demonstrates that support for research decreases when consumers’ trust in that environment decreases.^33^ Researchers have a responsibility to acknowledge the importance of earned trust as a critical pathway to patients’ research involvement.^34^

Participants offered suggestions to increase women Veterans’ inclusion and engagement in research communities. These suggestions can extend to both recruitment strategies for subjects and strategies to increase engagement of stakeholders/partners. The adoption of existing technologies to increase the visibility of research opportunities was emphasized across all participants, and aligns with other research.^35,36^ For instance, utilization of patient-facing portals of the electronic health record to disseminate real-time availability of local or national research opportunities, the adoption of patient research registries,^37^ and incorporating social media into recruitment methods have the potential to increase research visibility and to reduce the partnership gap between stakeholder/partners and researchers. Increasing participation and engagement hinges on researchers’ ability to build and sustain trust in their relationships with women Veterans, and to challenge the expectation that researchers arrive with a pre-determined need. Researchers may want to consider extending personally relevant information, such as the impetus for pursuing Veteran-engaged research; this can help to humanize research endeavors and build rapport. Anticipating women Veterans’ concerns about confidentiality and educating patients about how responses will not jeopardize care, nor disability ratings, may help to demystify how VA research operates. Additionally, the provision of study findings at the conclusion of studies can demonstrate genuine regard and underscore value toward study participants.^38^

Both women Veterans and WH-PCPs suggested that women Veterans’ mental health and trauma histories should be considered by researchers, raising important implications for the potential of adopting a trauma-informed approach to research engagement in VA. This is of particular importance to women Veterans given high reported rates of military sexual trauma (MST) history,^39^ and recent recommendations for the inclusion of trauma-sensitive care for women Veterans.^40,41^ Improving the likelihood of engagement in research (from subject to stakeholder) may require the scientific community to consider the adoption of trauma-informed principles that go beyond standard IRB regulations and procedures.^42^ In the same way the VA has entered a paradigm shift in trauma-informed care practices, research engagement efforts can benefit from sensitivity to the population with whom we seek partnership. Trauma-informed care delivery to patients has gained momentum as a best practice,^43,44^ but guidance is slow to take shape for trauma-informed principles related to research, despite the promise of such principles for mitigating challenges.

This study has limitations. First, the perspectives of women Veterans who utilize VA may differ from those who do not use VA. Second, women Veterans were recruited from VA health clinics in urban centers; perspectives in rural areas may differ. Additionally, findings are drawn from a small sample of women Veterans with diverse characteristics (e.g., age, military experience). MST histories were not sought, although these histories could influence results. Interviews were conducted with WH-PCPs and a small number of administrators, both of which lack demographic data, and do not necessarily reflect perspectives of PCPs outside of women’s health. Lastly, exploration of barriers and facilitators may reflect the limited scope resulting from women Veterans’ limited experience with research. Despite these limitations, this study is the first known exploration of research engagement from the perspectives of multilevel stakeholders; these perspectives combine to produce a dynamic exploration of barriers and facilitators related to research engagement.

Our exploration highlights the slow uptake of engagement as a practice, but also the demand for more inclusion across the spectrum of opportunities offered in health research. Our findings underscore the need for improved identification of engagement opportunities, as well as efforts to systematically, and culturally address multilevel barriers and facilitators to research engagement. Additionally, future research is needed to guide implementation methodologies for meaningful and ethical engagement practices. Efforts to mobilize diverse stakeholder populations (i.e., minorities, Veterans, women) ensure that health research is impactful and meaningful to the very population it aims to address.
